# Prevalence and Consequences of Swine Inflammation and Necrosis Syndrome (SINS) in French Herds

**DOI:** 10.3390/vetsci12090853

**Published:** 2025-09-03

**Authors:** Sandy Micout, Hervé Fortune, Gerald Reiner

**Affiliations:** 1 Swine Industry Expertise, ADM France, Route de Talhouët, 56250 Saint Nolff, France; herve.fortune@adm.com; 2 Clinic for Swine—Herd Health Management and Molecular Diagnostics, Department of Veterinary Clinical Sciences, Justus-Liebig-University Giessen, Frankfurter Strasse 112, 35392 Giessen, Germany; gerald.reiner@vetmed.uni-giessen.de

**Keywords:** inflammation, SINSs, animal welfare, swine, reproductive performance

## Abstract

A new, primarily endogenous syndrome affecting pigs from birth to fattening is associated with inflammation and necrosis of the tail, ears, teats, coronary band, heels, and claws. This disease negatively impacts the health, performance, and welfare of the animals. The present study aimed to determine the prevalence of the syndrome in typical herds in France and to investigate the associations between SINS, age and sex of the piglets and parameters of reproductive performance of the herds. Data from 2377 suckling piglets aged 1 to 4 days from 16 French farms were evaluated. SINS signs were found in 85.5% of the piglets. There were striking differences between farms, ranging from 68.5% to 95.6% affected piglets. The heels and coronary bands were the most commonly affected areas, at 61.4% and 58.6%, respectively. Alterations at the tail, ears, face, or teats were observed in 46% of the piglets. At least 3 body parts were affected per litter, and all examined body parts were affected on farms. The number of piglets born was significantly reduced in SINS. Screening was easy to implement and required only reasonable time management. It was also integrated into the zootechnical measures on the farms. The SINS lesion scoring system for piglets at birth appears to be a relevant indicator of animal health and welfare, as well as farm economic efficiency.

## Simple Summary

Swine Inflammation and Necrosis Syndrome (SINS) is a widespread disease in pigs, triggered by factors relating to their housing, feeding, management and genetics. Early detection of symptoms enables the rapid implementation of adapted care and environmental measures to maintain or restore health and performance. We present a binary matrix suitable for daily use on commercial farms. Significant differences in SINS prevalence were revealed between typical farms in a pig-producing region of France. Herds with a low prevalence of SINS were found to have superior reproductive performance. SINS lesions in piglets at birth, characterised by bristle loss, swelling, redness and necrosis in areas such as the tail, ears, teats and claws, appear to be a promising animal-based measure (ABM) that contributes to herd health, performance and animal welfare.

## 1. Introduction

Ensuring the health, welfare and performance of animals is a societal imperative for all professionals involved in animal care. A key to success is rapid detection and early intervention to prevent more serious disease, greater loss of performance, pain, suffering and harm. This is also the best way to meet consumer demand for uncontaminated food. This requires appropriate, clinically recognisable animal-based measures (ABMs) to detect milder forms early, before the more severe forms develop. Such measures are inflammations that occur as part of inflammation and necrosis syndrome, with loss of bristles, redness and mild swelling in the beginning [1].

Swine Inflammation and Necrosis Syndrome (SINS) describes a clinical situation in which signs of inflammation are observed in a herd, in litters and in individuals at the same time and in different parts of the body, which can lead to necrosis [2]. It begins with inflammatory hair loss, redness and swelling and, if the early signs are missed, can progress to exudation and necrosis [3].

In comparisons with piglets from the same litters living under the same environmental conditions, the association between the presence of signs of SINS and the presence of local inflammatory processes has been demonstrated by clinical [4,5], clinic-chemical [6], metabolomic [6,7], transcriptomic [7,8] and histopathological [4,9] methods as well as with massive shifts in liver metabolism towards an acute phase protein and inflammatory metabolism [7,8].

Based on these studies, the primary cause of this disease is currently thought to be an enrichment of microbial products (pathogen-associated molecular patterns, PAMPs) due to overloading of the gut and liver [2]. This distinguishes the syndrome from diseases that are essentially localised to one part of the body, such as panaritium of the claws, and biting of the tail, belly and ears, where typical wounds occur. In some studies, it is not clear whether only the individual conditions described were present because other parts of the body were not examined [10,11,12]. In the first hours of the piglet’s life, we note the presence of macrophages and lymphocytes in the intact epidermis of the tail at the level of the areas of inflammation [4]. These cells appear 4 to 7 days after the start of the inflammatory phenomenon [13], showing an endogenous origin of the phenomenon.

It is important to diagnose SINS because the problem is widespread [3,14,15,16] and because inflammation and necrosis generally cause pain, suffering and damage [17,18]. Due to the primarily endogenous origin, unilateral treatment and consideration of biting, unfavourable soil or treatment against *M. suis*, for example, may have only limited success if the intestine and liver are considered to be the triggering causes. The heritabilities observed from the female and the sire line is around 0.3 for inflammation of the tail, ears and claws [14], which shows that the changes that occur cannot be due to technopathy alone. Genetic effects of the sow and boar [3,5] and the involvement of the genome in various animal-specific predispositions have been demonstrated [8]. The controllability of the syndrome is not only understood in practice but has also been scientifically investigated and proven [9].

Studies by different authors from different countries show an increased incidence of inflammation and necrosis in pigs of different age groups (Table 1). There is considerable variability between studies, but on average between 30 and 40% of animals show signs of SINS in the parts of the body examined. The heels were the most severely affected, with an average of over 70%. The lowest prevalence was found in the face. The tail was affected in 11 to 84% of the animals. The studies by Reiner et al. and Leite et al. [3,14] (Germany), Fortune et al. [15] (France) and Koenders-van Gog et al. [1] (The Netherlands) were quite comparable in terms of prevalence. A study from Germany shows that weaners are more affected than suckling piglets, and both are more affected than fattening pigs [9].

Without diagnostics, the problem cannot be properly addressed or combated. Diagnosis is generally easy to perform by roughly cleaning the affected areas of the tail base, tail tip, ears, face, teats, coronary band, heels and claws and examining them for the inflammatory or necrotic changes described above [2]. Three-day-old piglets and weaners at around 30–35 days of age have been found to be particularly suitable age groups for diagnostics [2,14,15,16]. Studies are characterised by an attempt to minimise the effort required, especially under practical conditions. At the end of studies, there are always quantitative statements about SINS levels and SINS prevalence, which can be compared precisely within studies but only relatively between studies.

This study is based on the survey by Fortune et al. [15], in which the simple prevalence of SINS in suckling piglets in a pig-producing region in France was presented in the proceedings. The present study aimed to extend these results to include litter and herd prevalence and to investigate the effects of piglet age and sex, sow parity, and variability between farms. Additionally, this study aimed to investigate the association between SINS signs on body parts with different ground contact, as well as the relationship between the reproductive performance of the farms and the SINS grades of the piglets. This study also involved the use of a modified screening system that provided easier access to a larger number of animals under practical farm conditions.

**Table 1:** Prevalence of SINS signs in different body parts from different studies.

Country	Age	Tail Base	Tail Tip	Tail	Ears	Face	Teats	Coronary Bands	Heels	Study
GER	SP			11.3	0.5	16.3	4.3	54.1	73.4	[3]
GER	SP	48.7	32.2	(48.7) *	63.5		75.7	94.8	100	[9]
GER	WP	31.9	68.1	(68.1)	76.1		56.5	13.3	92.9	[9]
GER	FA	4.9	21.4	(21.4)	31.1		1	0	61.2	[9]
GER	NB	84.2	41.1	(84.2)	74	72.6	71.2	65.1	87.0	[4]
FRA	SP			23.5	10.8	16.9	12.9	58.6	61.4	[15]
GER	SP			16.9	19		14		43.5	[14]
NED	SP	25.2	19.1	(25.2)	14.5	6.2	20	34.8	64.1	[1]
Mean		39.0	36.4	37.4	36.2	28	32	45.8	72.9	

GER: Germany; FRA: France; NED: The Netherlands; SP; suckling piglets; WP: weaned piglets; FA: fatteners; NB: newborn piglets; *: no data of the complete tail available.

## 2. Materials and Methods

### 2.1. Study Design

This study was conducted in accordance with the Declaration of Helsinki. Ethical review and approval were waived because only non-invasive examinations were carried out as part of the herd examination. The only procedure was the visual collection of clinical scoring results, which was carried out as part of routine zootechnical measures for the piglets.

**Figure 1:**
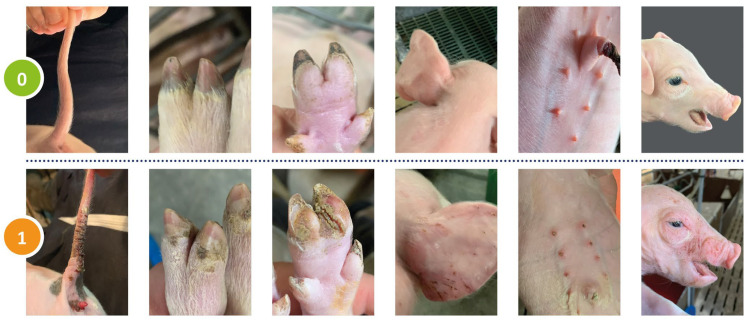
Clinical bimodal scoring of the tail, coronary bands, heels, ears, teats and face in 0 to 4 days old piglets. 0: no SINS lesions (**upper row**); 1 SINS lesions (**lower row**).

Clinical signs of SINS were scored in 2377 piglets from 16 randomly selected French pig herds at an age between 0 and 4 days. All piglets were scored by the same operator, who was assisted by a helper. Scoring was performed on animals with intact tails and prior to castration (Figure 1).

The study was conducted between September and December 2022. The farms had between 140 and 580 sows (mean ± SD: 326 ± 110), which were organised into 4, 5, 7, 10 and 21 batches on 3, 5, 2, 1 and 1 farms, respectively. The average batch size in this cohort was 53.3 ± 22.3 sows. On average, the number of sows per group was 81.6, 56, 42.1, 47.8 and 20 for farms managed in 4, 5, 7, 10 and 21 batches, respectively.

Eleven ± 2.9 sows per farm were randomly selected for this study (minimum: seven, maximum: 20), representing 21% of the sows available in the current batches.

The mean litter size was 15.92 ± 3.3 live piglets (range 7–25). Of these, 13.5 anatomically intact piglets per litter could be included in this study. A total of 160 litters were examined. It is important to note that each sow was used only once. Piglets were weaned at 21 days (62.5%) and 28 days (37.5%).

The farms were selected to be representative of the region’s current indoor farming system [19,20]. Pregnant sows were housed on slatted floors (14/16 herds) or straw floors (2/16 herds) and managed in dynamic groups (all parities and physiological stages in the same pen), static groups (same physiological stage and several parities in the same pen) or small groups (same physiological stage and parity in the same pen).

Feed was distributed according to a feeding curve adapted for genetics, housing, body condition, physiological stage, and feed formulation specifications. Regardless of their physiological stage, sows had free access to water. For 14 of these 16 farms, feed was provided by an industrial feed mill producer, while the remaining 2 farms produced feed on site according to French nutritional recommendations.

In the pregnancy unit, the feeding system was collective for small sow groups in eight of the farms and individual in the other batches, with an automated feeding system and ear tag reader adapted for dynamic and static sow management.

The ventilation system was passive in farms using straw and mechanical in farms with slatted floors. One week before farrowing, the sows were moved to the maternity unit and housed individually in crates fixed to a plastic slatted floor. One farrowing room was associated with one sow batch. In all farms, except for a few specific cases involving sows, farrowing was not induced by the use of prostaglandin drugs, and assistance for sows during farrowing was minimised.

During farrowing, the comfort of newborn piglets could be optimised using a heater light, a carpet or drying powder applied by farmers, or assistance at the teats for the first suckling.

Interventions on piglets were performed from 24 h after birth (iron injection and teeth grinding if necessary) to seven days (surgical castration in accordance with French legislation).

For this study, no changes to the routine management of sows or the farm were requested.

A total of 14 combinations of eight sow and six boar genotypes were used in the investigated herds. Due to the multiple combinations and the unresolvable interactions between farm management and genetics, both could not be considered in the statistical evaluation because these varied greatly across only a few farms. The numbers of piglets and farrowing performance associated with the examined litters, as well as the overall farrowing and reproductive performance of the farms (total live and stillborn piglets and piglets at weaning on days 21 and 28), obtained over the previous 12 months, were available for further analysis. Vaccine prophylaxis was tailored to each farm, resulting in a stable health status despite the presence of major contaminants such as PRRS, PCV2, Mycoplasma, and different species of E.coli in the region.

### 2.2. Clinical Scoring

In this study, inflammation and necrosis were recorded clinically with reference to [4], however with a more practical application of scoring. To ensure comparability with other studies, the piglets were scored on the day 3 of life. All piglets of selected litters were scored and litters that had more than 2 cross fostered piglets were excluded from the study.

In all previous studies, clinical signs were visible at this time, and the piglets were not yet as much exposed to environmental effects like weaners and fatteners.

Clinical alterations in the tail, the coronary bands, heels, ears, the teats as well as the face were assessed individually. The tail was scored for loss of bristles, swelling, redness, scab formation, rhagades, exudation, necrosis, bleeding, and ring-shaped constrictions. If any of these signs was present, the score was 1; if none of these signs was visible, the score was 0. Any other parts of the body were scored in a similar manner. Ears were scored for a shiny skin, the loss of bristles, necrosis and congested ear veins. The face was scored for the absence or presence of oedema at the eyelids and nose back. Teats were scored for swelling, reddening, scab formation, necrosis and congested blood vessels. Coronary bands and heels were scored qualitatively for any signs of inflammation. The score was 1 if at least one of the 4 claws was found positive. If any of the above signs of SINS were detected in an individual piglet, that piglet was considered positive, regardless of the type and severity of the signs. This was performed separately for the tail, ears, face, teats, coronary bands and heels. The scores for each body part were added to give the total SINS score, or the heels and coronary bands were omitted to give the SINS-feet score. The rationale for this approach was to be able to examine and quantify the influence of flooring conditions over time. Consequently, the total SINS score could range from 0 to 6 (6 body parts) and the SINS foot score from 0 to 4 (4 body parts). Subsequently, both values were recoded into bimodal variables (SINS01 and SINS-feet01), with 0 being retained and values from 1 to higher being recoded to 1. The score in question was utilised in the calculation of the prevalence of SINS and SINS-feet, among other metrics. This simplified methodology for recording SINS signs, compared to [4], was used to reflect the practical conditions in the herds and to achieve a compromise between detail and time required for the examination.

### 2.3. Statistical Analysis

Statistics were calculated using IBM SPSS version 27. The relationships between SINS scores and prevalence were analysed using a quadratic-plateau model with the nls function of R software (version 4.3.1). The prevalence of SINS signs on different parts of the body and the prevalence of piglets with SINS and SINS-feet were calculated using a general linear model for binary data (0/1). The effects of herd number (1–16), age at scoring (days 0–4), sex (entire male, female) and parity of the sow (1–13) were included as effects. The sow was included as a random effect. The statistical unit was the piglet. The statistical unit was the farm when analysing reproductive data. Finally, the 25th percentile of herds with the lowest and highest SINS scores were selected to examine SINS scores and reproductive performance in a comparison of the extremes (general linear model for binary data). Effects with a *p*-value ≤ 0.05 were treated as statistically significant.

## 3. Results

### 3.1. SINS in Individuals and Herds

**Figure 2:**
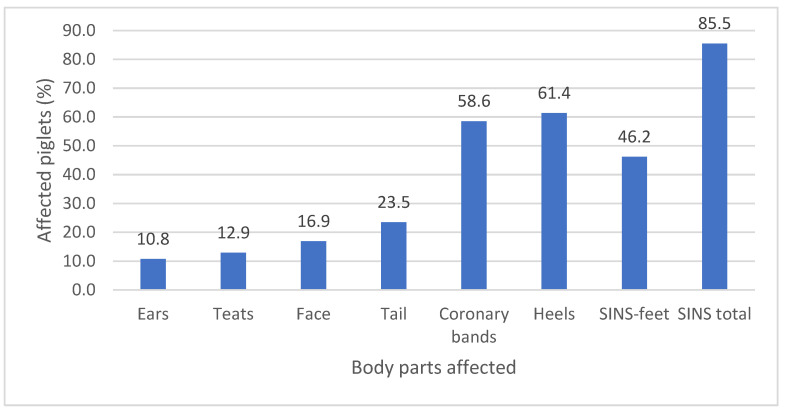
Prevalence of SINS signs on predisposed body parts of 2377 piglets scored in the first week of life (0–4 days post-partum) on 16 individual French farms.

A total of 2377 suckling piglets from 16 different farms were included in this study, with complete SINS scoring data available for all subjects. The mean lesion score recorded in this cohort was 1.8. The mean value of this ratio ranged from 1.1 to 2.6. Evidence of SINS was documented in a minimum of one anatomical region in 85.5% of the piglets examined. The highest number of lesions was found in the heels, affecting an average of 61.4% of piglets (Figure 2). The coronary band was affected in 58.6% of the piglets examined. The prevalence of SINS in other body parts ranged from 10.8% to 23.5%. Notwithstanding the exclusion of the coronary bands and heels, a proportion of 46.2% of the piglets exhibited signs of the condition. The present study found a strong positive correlation between total SINS scores and SINS-feet scores with a value of 0.791 and a *p*-value greater than 0.001.

**Figure 3:**
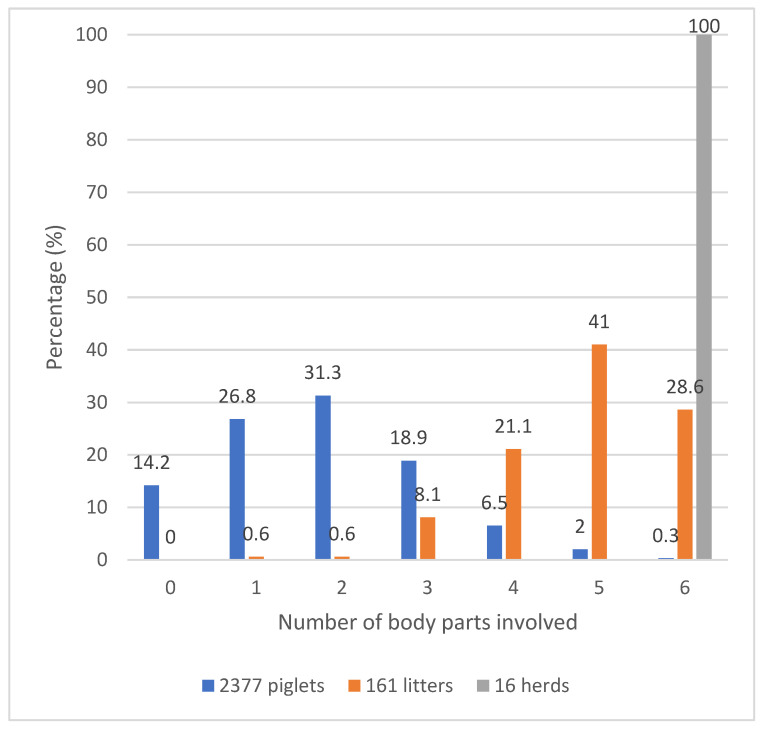
Percentage of piglets, litters and herds with 0 to 6 body parts simultaneously affected by SINS signs. The bars show the percentage of piglets (blue), litters (orange) and herds (grey) exhibiting SINS signs on 0, 1, 2, 3, 4, 5 or 6 body parts. The respective percentages are shown above the bars.

No evidence of SINS was identified in 14.5% of the examined piglets, with only one body part affected in 26.8% of cases. It is evident from Figure 3 that in at least 59% of the piglets, there was an occurrence of simultaneous involvement of two or more body parts. The majority of litters exhibited alterations to five body parts in a simultaneous manner. It was observed that no litters were entirely devoid of SINS. A further 0.6% of litters exhibited defects in one or two body parts. SINS manifested in all anatomical regions of all herds.

**Figure 4:**
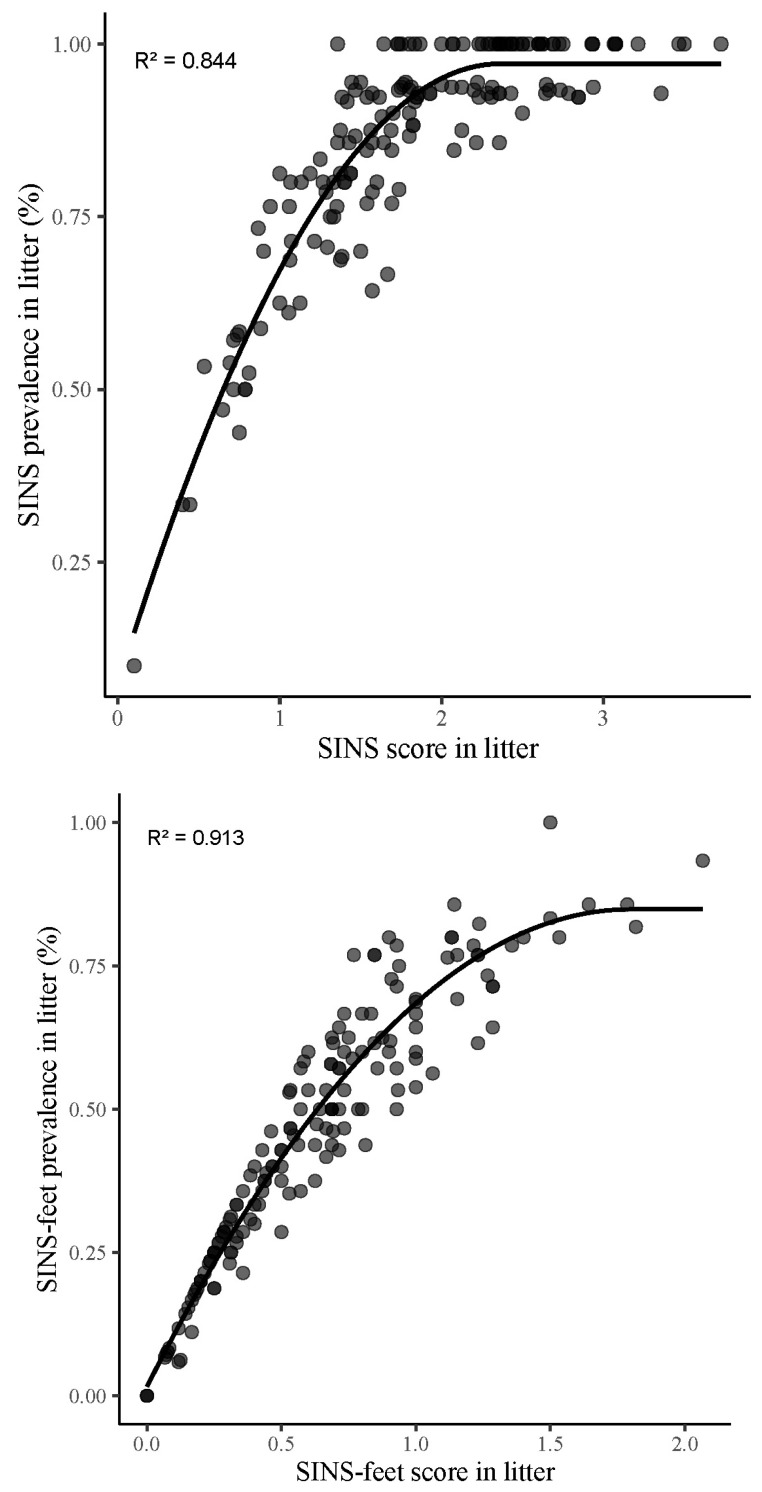
Correlation between SINS prevalence and SINS score in litters, including (**upper**) and excluding (**lower**) heels and coronary bands according to the plateau curvilinear model.

**Figure 5:**
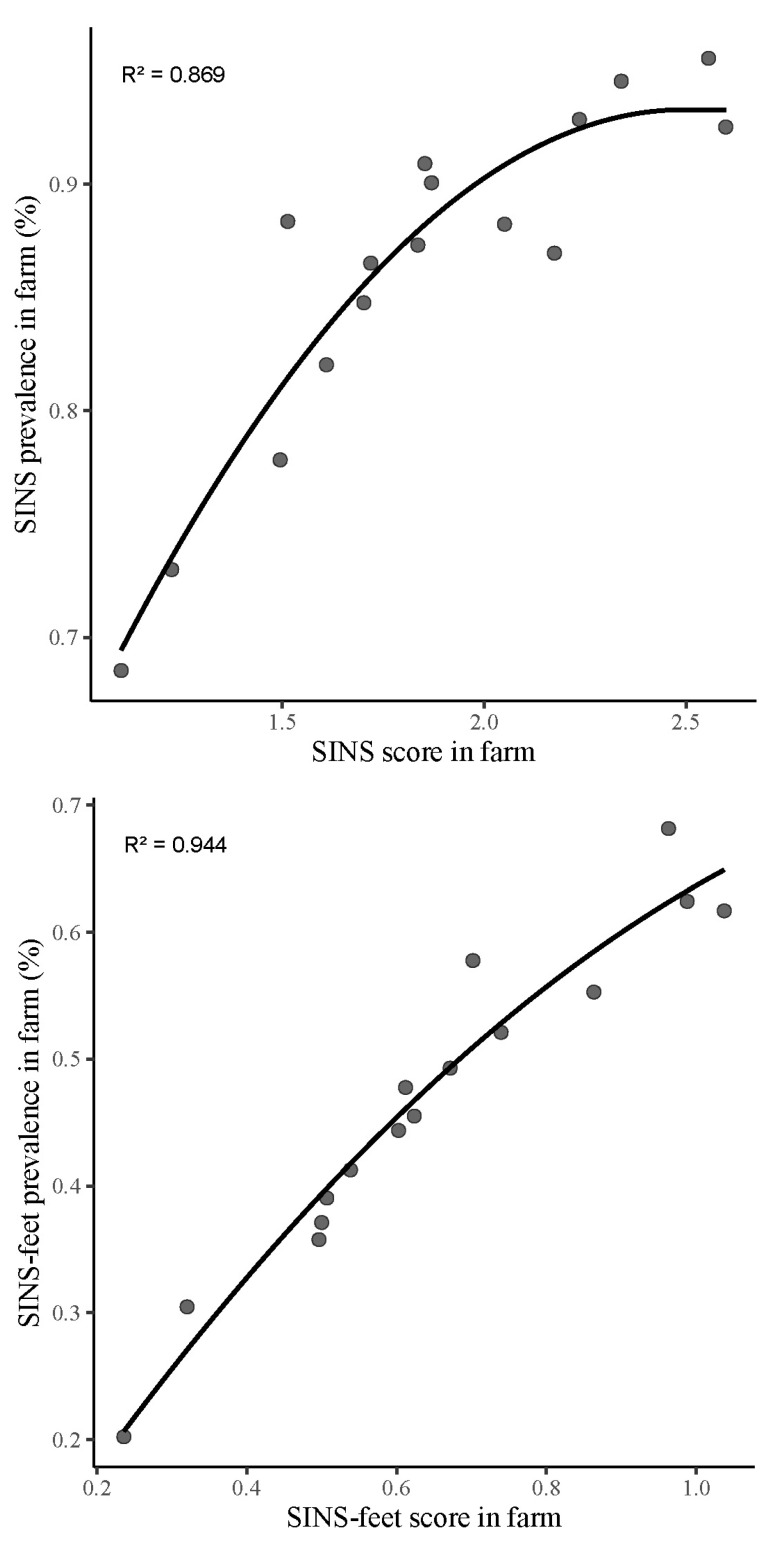
Correlation between SINS prevalence and SINS score in farms, including (**upper**) or excluding (**lower**) heels and coronary bands.

The prevalence of SINS signs in a litter was closely correlated with its average SINS score (r = 0.92, *p* < 0.001) (Figure 4). At the farm level, the correlation between SINS prevalence and SINS score was 0.96 (*p* < 0.001) (Figure 5). The present study found a strong correlation between score and prevalence with regard to SINS-feet, with a value of 0.93 (*p* < 0.001) at the litter level and 0.97 (*p* < 0.001) at the herd level. This finding suggests a consistent correlation between higher prevalence rates and higher SINS scores at both the litter and herd levels.

The presence of signs of SINS was identified in piglets across all herds, though the prevalence of clinical signs exhibited by individual farms exhibited significant variation. According to the herd, the percentage of piglets displaying SINS signs was as follows (mean ± SD (minimum–maximum)): Tail: 23% ± 12.6% (5–50.7%); ears: 12.7% ± 8.2% (3.8–26.4%); face: 15.3% ± 11.8% (3.9–45.4%); teats: 9.6% ± 5.2% (0.8–18.8%); coronary bands: 59.0% ± 14.1% (35.6–89.3%); heels: 69.3% ± 15.4% (34.5–91.8%). The percentages of SINS with and without consideration of the feet were 47.1% ± 13.1% (20.2–68.1%) and 86.5% ± 7.8% (68.5–95.6%), respectively.

Despite considerable variation in the involvement of the examined body parts among individuals, statistically significant differences were identified for all body parts when comparing the herds in the lowest and highest 25th percentile. In the top 25% of herds for SINS scores, tail scores were 165% higher and teat scores were 196% higher than in the bottom 25% of herds (Table 2). Furthermore, the face scores and SINS scores exhibited an increase that exceeded twofold. The most negligible disparities were observed for the heels (+71%), the coronary bands (+23%), and the total SINS score (+24%), yet these remained statistically significant.

### 3.2. Factors Associated with SINS

Despite the limited examination period of 0 to 4 days, a significant association was often observed between SINS scores and age (Table 3). There was a consistent increase in the prevalence of affected body parts from the day of birth to the 4th day of life. It was observed that the maximum values were only attained for tail, teats and SINS-feet on day 3. The course for the examined body parts was found to be parallel.

The prevalence of piglets exhibiting signs of SINS was found to be equivalent for both sexes; however, a significant disparity was observed in the SINS scores, with higher scores being recorded in females. This was particularly evident in the observation that the proportion of female piglets exhibiting signs of teat inflammation was approximately three times higher than that of male piglets (*p* < 0.001) (Table 4). The observed convergence in the prevalence of the sexes in the SINS score is due to the relatively high prevalence of affected coronary bands and heels overall, with slightly higher values recorded in males.

**Figure 6:**
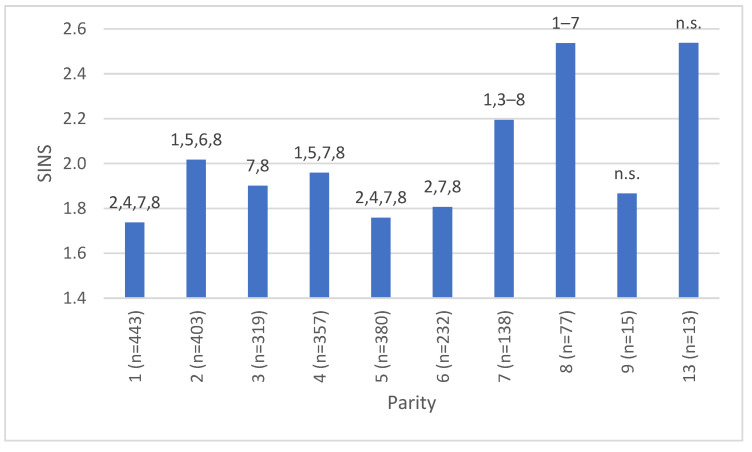
Association between SINS score and parity. Numbers above bars indicate significant differences between parities (*p* < 0.05); n = number of piglets with SINS scores.

A specific association was identified between the parity of the sows and the SINS score of the piglets (Figure 6). The SINS score of the piglets demonstrated an initial increase from parity 1 to 3, followed by a subsequent decrease to parity 5 and then a subsequent increase after parity 6. Following the seventh farrowing, a significant increase in the score was observed in comparison to previous parities (*p* < 0.001). Figure 6 also demonstrates the effects of parity 9 and 13, although it should be noted that the number of sows with these parities was limited.

With the exception of mortality rates, there were significant discrepancies in reproductive performance when comparing herds in the lowest and highest 25th percentile according to their SINS scores (Table 5). The herds demonstrating the lowest SINS prevalence exhibited a 2.1-fold increase in total born, a 1.6-fold increase in live-born, and a 1.3-fold increase in weaned piglets, in comparison to the herds exhibiting the highest SINS prevalence. Concurrently, however, the low-prevalence herds with the higher number of piglets also exhibited an additional 0.4 stillborn piglets.

Significant correlations were identified between the number of piglets (total born, born alive and weaned) and the number of piglets affected by SINS. The respective Spearman’s correlation coefficients were −0.715, −0.424, and −0.640, respectively, with the highest correlations obtained on the herd level (Table 6).

It has been demonstrated that an increase in SINS at the piglet, litter or herd level results in a decrease in the total number of piglets born and weaned. SINS scores demonstrated a higher degree of correlation than SINS prevalence.

**Figure 7:**
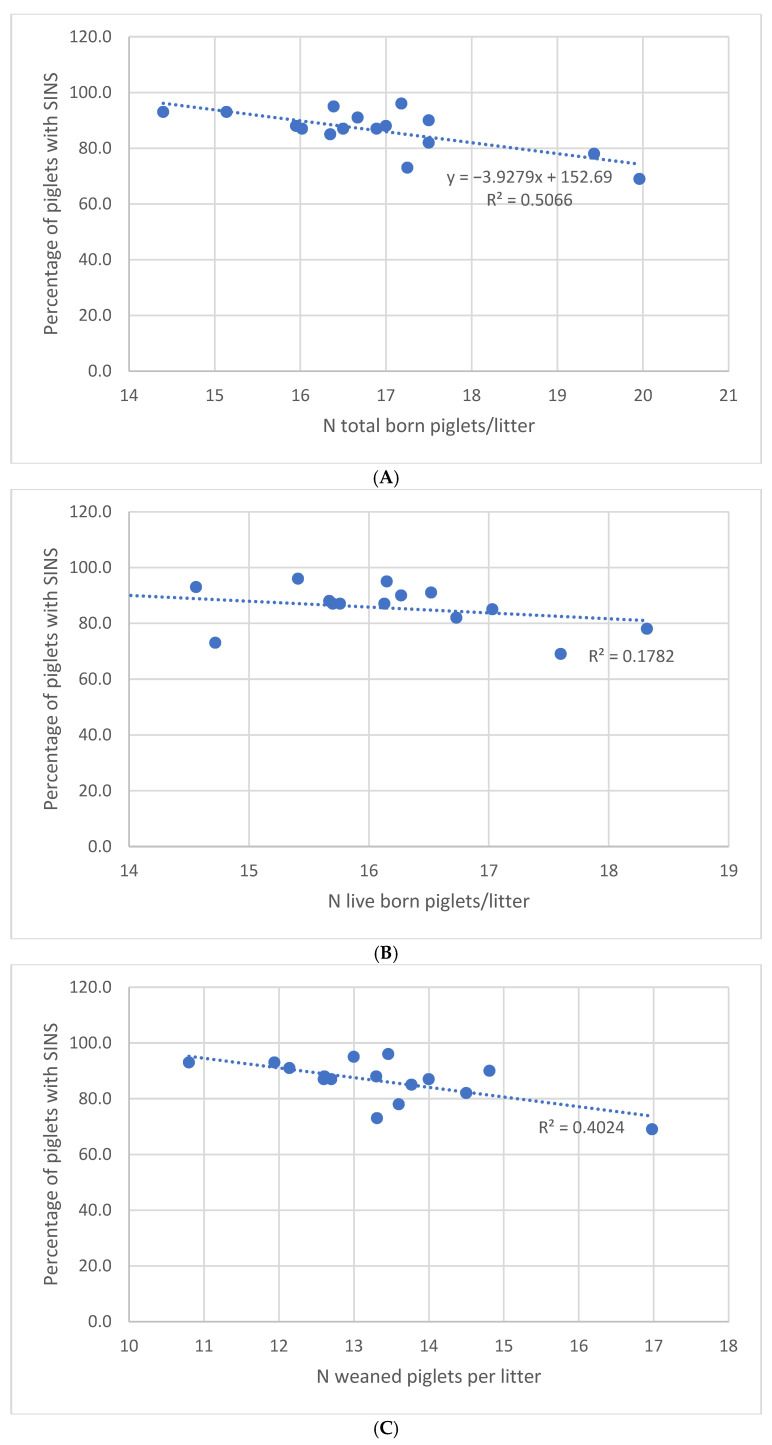
The relationship between the percentage of piglets affected by SINS and the total number of piglets born (**A**), the number of piglets live born (**B**) and the number of weaned piglets (**C**) per herd. The linear regression line and the coefficient of determination (R^2^) for each relationship are integrated.

This relationship is illustrated by the SINS prevalence per litter at the herd level (Figure 7), with an R^2^ = 0.40 between the number of piglets weaned per litter and the SINS prevalence of the farm. The linear regression indicates that 1% of SINS prevalence in litters induced a variation of 0.3 piglets weaned per litter.

## 4. Discussion

A novel syndrome (SINS) has been characterised by inflammation and necrosis observable in piglets from birth to higher age classes [2,3]. The syndrome appears to be widely distributed, including in France [1,3,14,15,16]. In the farms that were surveyed, 85.5% of the piglets that were examined exhibited signs of SINS [15]. These results are consistent with those reported in [1,3,14]. However, this value is lower than that reported in the results of Reiner et al. [9] and Kühling et al. [4]. A mere 14.5% of the piglets were found to be free of SINS, with 98.8% of the litters exhibiting at least three affected body parts and all body parts being affected in any of the herds. It was shown that inflammatory disorders in piglets around the time of birth may be prevalent among French farms also. However, a marked variation in the severity of problems experienced by different farms was observed. This is consistent with a recent study conducted in the Netherlands [1]. The observed variability between farms can be attributed to the numerous factors that can influence the qualitative and quantitative characteristics of the clinical manifestation [1,3,9].

For instance, it has been hypothesised that the ears are more severely affected when piglets’ thermoregulation is disrupted by high ambient temperatures. This assumption has been confirmed through thermal imaging [21]. Furthermore, it has been demonstrated that the condition of the heels and coronary bands is significantly impacted by flooring quality [10,11,12]. However, as demonstrated in the study by Kühling et al. [4], SINS lesions have been detected in the heels, coronary bands and claws of newborn piglets, when not yet exposed to the mechanical burden of the floor. The individual, floor-independent sensitivity of the heels and coronary bands was also confirmed based on the pronounced heritability of these traits [14]. The study by Koenders-van Gog et al. [1] also confirms the close relationship between body parts in close proximity to the floor and those that are more distant from it. Despite the temporal decline in this relationship, a substantial correlation persists between the initial SINS scores at the heels and coronary bands and their subsequent sensitivity to mechanical irritation from the floor [1]. In the present study, SINS scores with and without consideration of feet were found to be significantly associated with R^2^ = 0.799 (*p* < 0.001). These results suggest that the alterations observed in piglets at coronary bands and heels may be influenced by SINS and modified by the varying environment on different farms [1,2,9].

Besides different husbandry, feeding and management systems, virtually all herds examined comprised genetically distinct lineages. Considering the substantial variations in susceptibility to SINS among different lineages [1,3,5,9] and the relatively high heritability [14], it can be hypothesised that the observed disparities among farms are also attributable to these genetic differences. Due to the intricate interrelationships among husbandry practices, feeding methods, management strategies, and genetic factors on the farms, it was not possible to isolate and quantify the impact of individual factors on SINS in the present study. In order to ascertain the extent of the impact of these factors, it would be necessary to conduct investigations in select herds, ensuring that only the factors under scrutiny could be modified.

Despite the absence of definitive studies on this topic, it can be hypothesised that infectious diseases play a role in the pathogenesis of the condition [22]. A number of preliminary SINS studies were conducted in an SPF herd that was free of PRRSV, *Actinobacillus pleuropneumonia*, *Glaesserella parasuis*, scabies, PED, *Brachyspira hyodysenteriae*, *Lawsonia intracellularis*, *Brucella* sp., *Aujeszky’s disease*, Classical and African Swine Fever. At the time of this study, this herd was also free from disease by Influenza A and *Staphylococcus hyicus*. Nevertheless, SINS occurred to a considerable extent in newborns, suckling piglets and weaned piglets [4,5,6,8]. The exact health status of the herds of the present study was unknown. All herds were subject to their own vaccination regimes, and no clinical outbreaks of infectious diseases were evident at the time of the investigation.

The presence of lesions and inflammatory markers in piglets at birth suggests an endogenous disorder initiated by the sows in pregnancy and transmitted to the piglets before birth [4]. Based on this, SINS lesions in piglets at birth appear as quality criteria related to the overall management of sows, as well as a diagnostic tool associated with nutritional, housing, hygiene, and health patterns of the animals [9].

A comparison of the zootechnical results from different farms reveals that farms with a low SINS prevalence were also more efficient in terms of total piglets born (18.6 vs. 16.5, *p* < 0.001) and born alive (16.9 vs. 15.3, *p* < 0.001). Furthermore, an average of 1.3 additional piglets were weaned per sow on low-prevalence farms (*p* = 0.002). However, a significantly higher number of stillbirths was observed on these low-prevalence SINS farms (8.7% vs. 7.2% of total born piglets). At this juncture, a hypothesis can be postulated that a prolonged birth duration in conjunction with higher prolificacy could be responsible for these increased rates of stillbirths [23], though further investigation is required to substantiate this claim.

Concurrently, piglets on farms with elevated SINS levels did not demonstrate reduced mortality rates until weaning, despite being subjected to reduced competition for teats and diminished colostrum intake [23,24]. The negative correlation between the prevalence of SINS and the number of weaned piglets per litter also emphasises the utility of lesion assessment as a tool for predicting the extent to which the needs of piglets are being met, thereby optimising the technical performance of farms [1]. For instance, a one-point reduction in the prevalence of SINS at farm level resulted in 0.3 more weaned piglets per litter. This corresponds to an estimated €65,000 in turnover for a 500-sow farm in the French context as of April 2025 (price paid: €1.90/kg carcass; 97 kg/carcass) [25]. This appears to be a relevant factor in achieving optimal economic efficiency in an agricultural context.

Furthermore, a statistically significant difference was observed in the SINS score of piglets from sows with a parity over seven in comparison to those from other sows (*p* = 0.009). It is evident that comparative results are not available in the extant literature. In this segregation, no significant difference was observed between lesions in parity groups 1–3 and 4–6. However, when litters from parity 1 to 4 are considered, the SINS score comparison of consecutive parities indicates that parity 2 is more sensitive to lesions. However, given the trajectory of the curve and the prevailing practices in the management of underperforming sows with regard to their prolificacy, milk production, and weaning ability, a significant proportion of young females are culled at parities 2–3 [26]. In consideration of the relationship observed in this study between prolificacy, the number of piglets weaned per litter, and SINS, it can be hypothesised that anticipated sow removal based on these production criteria induces indirect selection against SINS. This hypothesis provides a rationale for the decrease in SINS scores at parities 4, 5, and 6. Nevertheless, this beneficial effect of selection is likely to be eclipsed by a further increase in the age of the sow. As is well documented, the quality of sows declines with age due to mastitis, injuries, claw damage, etc., and this seems to increase the prevalence of SINS in piglets [9,26]. This could be one of the factors contributing to the observed deterioration from the seventh litter onwards. It is important to note that higher parities are only achieved by sows of a particularly superior quality. However, the number of such sows available was insufficient to allow for reliable statements to be made on this matter. In order to confirm or refute this hypothesis, it is necessary to characterise the SINS score of the last litter of the culled sow, with particular reference to the anticipated culled sow. The objective is to optimise economic efficiency by avoiding the premature removal of sows before they reach their third parity [27]. This is associated with technical and health performance indicators.

In order to assess the importance of SINS in a herd with regard to welfare, zootechnical performance and economics, and to implement appropriate responses, it is necessary to have a simple, reliable and easy-to-implement diagnostic indicator. Thus, one of the purposes of this study was to apply a qualitative binary method for SINS lesion scoring to validate its applicability in routine use on commercial farms with regard to practicability and differentiation between herds.

For widespread dissemination, the method must be: (i) easy to use, requiring limited training time and operator qualification standards; (ii) non-restrictive in terms of farm organisation, requiring a short amount of time and personnel; and (iii) robust and reliable and linked to technical and/or health performance indicators.

The present study considerably extended the prevalence of SINS in suckling piglets in a specialised pig production region in Brittany, France, as published by Fortune et al. [15]. Additionally, the prevalence of litters and herds was considered, as were the effects of piglet age and sex, sow parity, variability between farms, and the farms’ reproductive performance. This included 160 litters from 16 typical French herds. Two people (one assistant and one assessor) conducted the assessment on piglets with different ages between 0 and 4 days old, where each piglet was measured only once. Based on this organisation, the average time required to assess a litter was seven to eight minutes, including moving from litter to litter, grouping the piglets, recording litter information, and scoring lesions.

Based on experience, the time required for scoring appears to be an important parameter. To minimise bias (false positives), the time spent per body part was limited to 3 s, and 90–120 min was allotted to assess all litters. This seems to correspond to the maximum attention span of a scoring operator, considering cognitive, physical, and visual fatigue [28].

The qualitative, binary matrix’s focus on the six body parts most susceptible to SINS lesions allows for the assessment of a sufficient number of piglets and litters within these time constraints. The lesions observed on the animals at the time of scoring were consistent with the descriptions of Reiner et al. [9] and Koenders-van Gog et al. [1]. Scoring lesions at 3 to 4 days of age appears to be a good compromise between applicability of the method (e.g., ergonomics and integration into other routine tasks) and appearance of lesions [29]. Additionally, lesion assessment time can be optimised for routine implementation when carried out simultaneously with other piglet care, seamlessly integrating this information collection within a farm’s organisational routine.

The simplified data collection method used in this study was easy to implement when examining the piglets. This allowed for good comparability with other studies based on prevalence. However, it was not possible to record and analyse the SINS grades in a comparable way. In this study, SINS scores ultimately measured the number of body parts under investigation affected per animal. Considering that SINS begins with mild signs, such as loss of bristles, redness, and swelling, and that the majority of suckling piglets in previous studies showed such mild signs, while only about 1–4% were affected by exudation, haemorrhage, or necrosis, the importance of recording individual findings separately for a more precise characterisation of the animals and more meaningful comparisons becomes clear [4,5]. A recent study by Lösel et al. [16] on SINS, which included 13,000 1- to 5-day-old suckling piglets from 16 Bavarian herds in Germany, revealed the following signs: loss of bristles (28.1%), swelling (28.2%), redness (41.1%), exudation (13.7%), and necrosis of the tail (2.8%). These findings provide insight into the severity of the disease. Kuehling et al. [5] examined litters from the most SINS-sensitive boars and found necrosis in 16.7% of piglets at the tail base, 2.2% at the tail tip, 61.4% at the teats, and 1.4% on the face. A disadvantage of prevalence compared to scores is that prevalence ends at 100%, whereas scores can be open-ended. More practical solutions for standardising SINS scores that take feasibility into account need to be found. However, it has been shown that, despite different SINS scores, relative comparisons between trials and absolute comparisons of treatment groups, genetics, etc., within trials are possible.

Finally, the positive correlation observed between SINS score and SINS prevalence in litters (R^2^ = 0.793, *p* < 0.001) indicates that a higher SINS prevalence is associated with a higher number of lesions, thereby characterising the severity of field cases for a routine usage in commercial farms.

The syndromic nature of SINS was confirmed. In particular, several body parts were always affected simultaneously, especially at the litter and herd levels. This is consistent with the high degree of correlation in the progression of different body parts, which would not be expected if the signs were caused by different factors. These findings corroborate the studies of Reiner et al. [3,9] and Kühling et al. [4,5]. As Koenders-van Gog et al. [1] previously described, the present study also revealed a significant sex effect, particularly regarding alterations in the teats, affecting female piglets more than male piglets.

The SINS score in piglets around the time of birth appears to be a relevant indicator of animal health and welfare, as well as the economic efficiency of farms, and SINS lesions could serve as an indicator of the adequacy of the relationship between pigs and their environment, as well as the overall farm management, regardless of the stage of growth.

## 5. Conclusions

The results of this study show that SINS lesions can be observed in French herds. Depending on their prevalence, these lesions can impact the technical and economic performance of farms. Underlying inflammatory lesions can be considered clinical signs of metabolic disorders, making pigs more sensitive to their environment. The binary matrix is simple to use, requires minimal time, and provides a useful indication of the general health, technical, and animal welfare status of the farm. This makes it a valuable management tool for commercial farms, in line with the HACCP (Hazard Analysis and Critical Control Points) concept.

## Figures and Tables

**Table 2:** Signs of SINS in piglets from 25% herds with lowest and 25% of herds with highest SINS prevalence.

	SINS Low ^1^ n = 635	SINS High n = 568	*p* ^2^	Increase ^3^
Tail (%)	13.8	36.6	<0.001	+165.2%
Ears (%)	7.7	12.3	0.007	+59.7%
Face (%)	12.1	26.4	<0.001	+118.2%
Teats (%)	5.3	15.7	<0.001	+196.2%
Coronary bands (%)	51.8	63.9	<0.001	+23.4%
Heels (%)	44.3	75.7	<0.001	+70.9%
SINS-feet (%)	30.8	62.3	<0.001	+102.3%
SINS total (%)	75	93.1	<0.001	+24.1%

^1^: Percentage of piglets on the 25% farms with lowest SINS prevalence; ^2^: general linear model for binary data; ^3^: increase from SINS low farms to SINS high farms by %.

**Table 3:** Association between the piglets’ age and the prevalence of SINS.

Day	0 (n = 282)	1 (n = 1022)	2 (n = 653)	3 (n = 347)	4 (n = 73)	*p* ^3^
Tail (%) ^1^	14.3 a ^2^	20.8 b	23.3 c	29.8 d	15.6 acd	0.004
Ears (%)	5.7	5.7	10.9	16.6	16.6	0.02
Face (%)	6.9 a	10.1	11.4	15.9 b	16.3	0.089
Teats (%)	6.2 a	6.6 a	9.5	12.6 b	5.4	0.065
Coronary bands (%)	55.8	55.3 a	64.7 b	63.8 b	62.8	0.024
Heels (%)	58.5 a	61.1 a	75.9 b	75.3 b	78.1 b	<0.001
SINS-feet (%)	41.1 a	44.4 b	47.8 c	52.7 d	45.2 abcd	<0.001
SINS total (%)	85.8	83.2	88.4	88.5	87.7	n.s.

^1^: Percentage of piglets affected; ^2^: different letters indicate significant difference at *p* < 0.05); ^3^: general linear model for binary data.

**Table 4:** Association between piglets’ sex and prevalence of SINS.

	Male (n = 1117)	Female (n = 1095)	*p* ^2^
Tail (%) ^1^	22.5	24.0	n.s.
Ears (%)	8.7	11.1	0.036
Teats (%)	6.7	19.0	<0.001
Coronary bands (%)	60.9	56.9	0.031
Heels (%)	60.0	59.8	n.s.
SINS (%)	85.6	84.8	n.s.
SINS-feet (%)	39.7	50.4	<0.001
SINS Score (mean ± SE)	1.73 ± 1.16	1.88 ± 1.3	0.005 ^3^
SINS-feet Score (mean ± SE)	0.52 ± 0.74	0.71 ± 0.85	<0.001 ^3^

^1^: percentage of piglets affected; ^2^: generalised linear model for binary data; ^3^: generalised linear model for metric data.

**Table 5:** Associations of extreme herds with respect to SINS with reproductive performance.

	SINS Low Herds (n = 30 Litters)	SINS High Herds (n = 40 Litters)	*p* ^1^
Total Born piglets	18.6 ± 0.20	16.5 ± 0.24	<0.001
Live born piglets	16.9 ± 0.17	15.3 ± 0.2	<0.001
Stillborn piglets	1.63 ± 0.05	1.19 ± 0.06	<0.001
Weaned piglets	14.5 ± 0.25	13.2 ± 0.30	0.002
Mortality Live born to weaning	14.3 ± 0.86	14.2 ± 1.02	n.s.

^1^: generalised linear model for metric data.

**Table 6:** Spearman’s correlation coefficients of SINS score and SINS prevalence, with (SINS) and without (SINS-F) consideration of coronary bands and heels) with the number of piglets.

Trait	Level	SINS	SINS01	SINS-F	SINS-F01
Total born piglets/litter	Piglet (n = 2377)	−0.237 **	−0.161 **	−0.168 **	−0.156 **
	Litter (n = 161)	−0.398 **	−0.325 **	−0.334 **	−0.327 **
	Herd (n = 16)	−0.687 **	−0.715 **	−0.628 **	−0.608 *
Live born piglets/litter	Piglet	−0.250 **	−0.168 **	−0.176 **	−0.160 **
	Litter	−0.197 *	−0.180 *	−0.166 *	−0.170 *
	Herd	−0.545 *	−0.424	−0.556 *	−0.498 *
Weaned piglets/litter	Piglet	−0.230 **	−0.142 **	−0.170 **	−0.145 **
	Litter	−0.389 **	−0.289 **	−0.337 **	−0.304 **
	Herd	−0.668 **	−0.640 **	−0.638 **	−0.571 *

SINS: SINS score including all examined body parts; SINS01: binary mode of SINS: SINS score of 0 was recoded to 0, any SINS score of 1 or higher was recoded to 1; SINS-F: SINS score including all examined body parts, except coronary bands and heels; *: *p* < 0.05; **: *p* < 0.01.

## Data Availability

The datasets used and analysed during the current study are available from the corresponding author on reasonable request.

## Abbreviations

The following abbreviations are used in this manuscript:

SINS	Swine Inflammation and Necrosis Syndrome
